# Quantification of IgM molecular response by droplet digital PCR as a potential tool for the early diagnosis of sepsis

**DOI:** 10.1186/cc13910

**Published:** 2014-06-06

**Authors:** Eduardo Tamayo, Raquel Almansa, Elena Carrasco, Ana Ávila-Alonso, Ana Rodríguez-Fernández, John Wain, María Heredia, Esther Gomez-Sanchez, Susana Soria, Lucia Rico, Verónica Iglesias, Ángel Martínez-Martínez, David Andaluz-Ojeda, Jose Ignacio Gómez Herreras, Jose Maria Eiros, Jesús F Bermejo-Martin

**Affiliations:** 1 Servicio de Anestesiología y Reanimación, Hospital Clínico Universitario-SACYL, Avda Ramón y Cajal 3, 47005 Valladolid, Spain; 2 Immunity, Risk of Infection and Sepsis group (IRIS), Hospital Clínico Universitario de Valladolid, Avda Ramón y Cajal 3, 47005 Valladolid, Spain; 3 Unidad de Apoyo a la Investigación, Hospital Clínico Universitario de Valladolid, IECSCYL-SACYL, Avda Ramón y Cajal 3, 47005 Valladolid, Spain; 4 Servicio de Microbiología, Hospital Clínico Universitario-SACYL, Avda Ramón y Cajal 3, 47005 Valladolid, Spain; 5 Norwich Medical School, University of East Anglia, Norwich, Norfolk NR4 7TJ, UK; 6 Unidad de Cuidados Intensivos, Hospital Clínico Universitario-SACYL, Avda Ramón y Cajal 3, 47005 Valladolid, Spain; 7 Hospital Clínico Universitario-SACYL, Avda Ramón y Cajal 3, 47005 Valladolid, Spain

## 

Evaluation of host immune response to infection at the molecular level is a promising avenue to obtain diagnostic and prognostic tools for the clinical management of patients with sepsis. A recent report from Cajander and colleagues [1] has shown the potential of HLA-DR mRNA quantification by real-time PCR as a biomarker of immunosuppression in these patients. IgM is the first immunoglobulin produced in response to infection. In a pilot study, we have employed a next generation quantitative PCR method (nanoliter-sized droplet technology paired with digital PCR (ddPCR)) for detecting the early transcriptomic response of IgM in blood from patients with sepsis. Approval for the study protocol for both scientific and ethical aspects was obtained from the Committee for Clinical Research of Hospital Clínico Universitario, Valladolid, Spain. Written informed consent was obtained directly from each patient or a legal surrogate. The target gene transcript was IGHM, which encodes the constant region of the mu heavy chain, which defines the IgM isotype [2]. In blood, the cells producing IgM transcripts are B lymphocytes expressing CD20 [3], which was employed as housekeeping gene.

Fifty-five patients with sepsis were recruited, 42 of them presenting criteria of septic shock (Additional file 1). Septic patients were predominantly older males (n = 40, 72.7%; mean age 72 years (standard deviation 9.3)). Mean Sepsis-related Organ Failure Assessment score was 8.4 (standard deviation 2.9). Overall ICU mortality was 34%. Emergency surgery was needed in 54% of cases, with cardiac and abdominal surgery the most frequent (45% and 40%, respectively). Respiratory infection was present in 34.5% of the cases. Frequency of abdominal infection was also 34.5%. Gram-negative bacteria were the most frequent isolated (56. 4% of cases). In parallel, we recruited 20 patients with post-surgical systemic inflammatory response syndrome (SIRS) and 15 healthy controls.

Compared to real-time quantitative PCR, ddPCR offers greater precision and reproducibility [4]. ddPCR allowed us to identify the presence of an early molecular response of IgM in the blood of patients with sepsis compared with healthy controls and patients with SIRS. This response was more intense in the most severe patients (Figure 1). When accuracy and the predictive value of the IGHM/CD20 ratio for diagnosing sepsis were analyzed, the area under the receiver operating characteristic curve was 0.72 (95% confidence interval 0.60 to 0.85; *P* = 0.003; Figure 1). In conclusion, quantification of IgM response at the transcriptomic level by ddPCR represents a promising approach for the early detection of sepsis.

**Figure 1:**
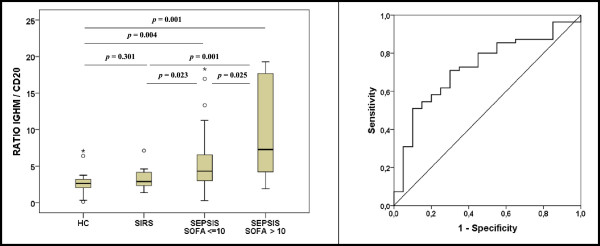
**IgM transcriptomic response in the presence/absence of sepsis.** Left: comparison of immunoglobulin (Ig)M transcriptomic response between groups. HC, healthy control (n = 15); SIRS, systemic inflammatory response syndrome (n = 20); SEPSIS SOFA < =10, Sepsis with Sepsis-related Organ Failure Assessment score ≤10 (n = 39); SEPSIS SOFA >10, sepsis with SOFA score >10 (n = 16). Results for the ratio are expressed as (Copies IGHM/Copies CD20). Right: receiver operating characteristic curve of IGHM/CD20 predicting presence of sepsis in the comparison (sepsis versus SIRS). For this comparison septic patients were considered as a single group.

## Abbreviations

ddPCR: droplet digital PCR; Ig: Immunoglobulin; PCR: Polymerase chain reaction; SIRS: Systemic inflammatory response syndrome.

## Competing interests

The authors declare that they have no competing interests.

## Authors’ contributions

ET, JW, and JFBM helped with the study design, provided a critical review of the results and participated in article writing; RA, AAA, ARF, DAO, JIGH, and JME analyzed the data and participated in article writing; EC, MH, EGS, SS, and AMM recruited the patients, provided a critical review of the results and participated in article writing; LR and VI performed the molecular works and provided a critical review of the results. All authors read and approved the final manuscript.

## Supplementary Material

Additional file 1 Additional file 1 is the supplemental methods. Click here for file
