# Supplementary material for: Quantification of IgM molecular response by droplet digital PCR as a potential tool for the early diagnosis of sepsis
Source: Crit Care. 2014 Jun 6;18(3):433. doi: 10.1186/cc13910 (PMC4075261; doi:10.1186/cc13910)
Supplement: Additional file 1 — is the supplemental methods. [file cc13910-S1.doc]

**Methods**

**Patient population and Inclusion criteria:** Adult patients (> 18 yr old) fulfilling the criteria of sepsis (n = 55) or systemic inflammatory response syndrome (SIRS) (n = 20) were prospectively recruited from the Reanimation Unit of the “Hospital Clínico Universitario de Valladolid” in Spain from January to December 2012. Recommendations of the American College of Chest Physicians/Society of Critical Care Medicine Consensus Conference were followed to define sepsis and SIRS [1]. Fifteen healthy controls of similar age and sex composition were recruited between workers of our Hospital for comparison purposes.

**Microbiology:** Standard cultures in biological samples guided by the presumptive source of the septic insult were performed to assess the presence of bacterial and

fungal infection. Potentially contaminant microorganisms were not considered.

**RNA samples:** a sample of 2.5 mL of blood was collected by using PaxGene (BD) venous blood vacuum collection tubes in the first 24 hours following diagnosis of sepsis or SIRS, and also from controls. Total RNA was extracted from blood samples using the PAXgene Blood RNA System (PreAnalytix, Hombrechtikon, Switzerland). The evaluation of quantity and quality was performed by spectrometry (NanoDrop ND1000, NanoDrop Technologies, Wilminton, Delaware USA) and RNA Experion Bioanalyzer (BioRad, California USA).

**IGHM and CD20 mRNA quantification:** Primers and probes for quantifying mRNA from both genes were designed by using Primer Blast, and were purchased to IDT (Iowa, USA). Probes were double-quenched internally with internal ZEN Quencher and 3′ Iowa Black® Fluorescent Quencher (IBFQ). Fluorofores for IGHM and CD20 were FAM and HEX (respectively). Sequences were as follows: IGHM (immunoglobulin heavy constant mu): forward primer (Sequence 5'-3'): AGAAGTATGTGACCAGCGC; reverse primer: CATTCCTCTTCGGACACGG; internal oligo: CCGGTACTTCGCCCACAGCATCCT. CD20: forward primer: AGAGTTACCACACCCCATGA; reverse primer: GCCTAGAGTGGGAGTTAGGA; internal oligo: GGGAAGCTCTAAATAGCCAACACCCATCT. Briefly, cDNA was generated from each sample on a BioRad C1000 thermal cycler starting from 500 ng of mRNA by using iScript Advanced kit from Biorad. The obtained cDNA was further diluted (1/25), and 5 μl of the diluted cDNA were assayed by ddPCR for quantification of IGHM and CD20 transcripts. ddPCR was performed using the Bio-Rad QX100 ™ Droplet Digital ™ PCR system, ddPCR supermix, and Bio-Rad standard reagents for droplet generation and reading. The process consists of the following steps: generating droplets, performing PCR, reading droplets, and analyzing the results. Each sample was analyzed by duplicate. Mean of each duplicate was employed in the statistical analysis.

**Statistical analysis:** comparison of IGHM/CD20 between groups was performed using the Mann Whitney U test. The accuracy and the predictive values of IGHM/CD20 for diagnosing sepsis were studied by calculating the area under the receiver operating characteristic curve (AUROC). Statistical significance was fixed at level (*p* < 0.05).
